# Interaction of childhood urbanicity and variation in dopamine genes alters adult prefrontal function as measured by functional magnetic resonance imaging (fMRI)

**DOI:** 10.1371/journal.pone.0195189

**Published:** 2018-04-10

**Authors:** Jessica L. Reed, Enrico D’Ambrosio, Stefano Marenco, Gianluca Ursini, Amanda B. Zheutlin, Giuseppe Blasi, Barbara E. Spencer, Raffaella Romano, Jesse Hochheiser, Ann Reifman, Justin Sturm, Karen F. Berman, Alessandro Bertolino, Daniel R. Weinberger, Joseph H. Callicott

**Affiliations:** 1 Clinical and Translational Neuroscience Branch, Division of Intramural Programs, National Institute of Mental Health, National Institutes of Health, Bethesda, Maryland, United States of America; 2 Interdisciplinary Program in Neuroscience, Georgetown University Medical Center, Washington, District of Columbia, United States of America; 3 Experimental Therapeutics & Pathophysiology Branch, Division of Intramural Programs, National Institute of Mental Health, National Institutes of Health, Bethesda, Maryland, United States of America; 4 Lieber Institute for Brain Development, Johns Hopkins Medical Campus, Baltimore, Maryland, United States of America; 5 Psychiatric Neuroscience Group, Department of Basic Medical Science, Neuroscience and Sense Organs, University of Bari Aldo Moro, Bari, Italy; 6 Departments of Psychiatry, Neurology, Neuroscience and the McKusick-Nathans Institute of Genetic Medicine, Johns Hopkins University School of Medicine, Baltimore, Maryland, United States of America; Maastricht University Medical Center, NETHERLANDS

## Abstract

Brain phenotypes showing environmental influence may help clarify unexplained associations between urban exposure and psychiatric risk. Heritable prefrontal fMRI activation during working memory (WM) is such a phenotype. We hypothesized that urban upbringing (childhood urbanicity) would alter this phenotype and interact with dopamine genes that regulate prefrontal function during WM. Further, dopamine has been hypothesized to mediate urban-associated factors like social stress. WM-related prefrontal function was tested for main effects of urbanicity, main effects of three dopamine genes—catechol-O-methyltransferase (*COMT*), dopamine receptor D1 (*DRD1*), and dopamine receptor D2 (*DRD2*)—and, importantly, dopamine gene-by-urbanicity interactions. For *COMT*, three independent human samples were recruited (total n = 487). We also studied 253 subjects genotyped for *DRD1* and *DRD2*. 3T fMRI activation during the N-back WM task was the dependent variable, while childhood urbanicity, dopamine genotype, and urbanicity-dopamine interactions were independent variables. Main effects of dopamine genes and of urbanicity were found. Individuals raised in an urban environment showed altered prefrontal activation relative to those raised in rural or town settings. For each gene, dopamine genotype-by-urbanicity interactions were shown in prefrontal cortex–*COMT* replicated twice in two independent samples. An urban childhood upbringing altered prefrontal function and interacted with each gene to alter genotype-phenotype relationships. Gene-environment interactions between multiple dopamine genes and urban upbringing suggest that neural effects of developmental environmental exposure could mediate, at least partially, increased risk for psychiatric illness in urban environments via dopamine genes expressed into adulthood.

## Introduction

As people live in ever-denser population centers, the implications of urbanization for health and well-being remain unclear. Urban environments carry both benefits and risks [1]. Urban upbringing and urban living (“urbanicity”) offer benefits from increased productivity and prosperity, higher wages, and increased access to cultural resources [2]. However, city life carries disadvantages like increased crime [3], over-population [4], psychiatric symptoms from crowd exposure [5], and demanding social environments [2]. Urbanicity, related to poorer mental health, increases risk for anxiety and mood disorders [6], and a doubling of the lifetime prevalence of schizophrenia with an associated population relative risk larger than family history [7]. Known for some time, environment and gene-environment (GxE) interactions alter brain development in experimental animals [8–9]. These effects have ramifications within the brain including neurogenesis, neurotransmission, brain size, and cognition [10–13].

Environmental factors likely influence heritable complex human traits, from temperament and cognition to psychiatric illness risk [14–16]. For example, allelic variation in the serotonin transporter gene *SLC6A4* increases susceptibility to depression in adults exposed to stress during early development [17]. In addition, obstetrical complications interact with genes implicated in hypoxic-ischemic tissue injury increasing risk for adult schizophrenia [18]. Neuroimaging suggests that the human brain is sensitive to environmental influences, as evidenced by London taxi drivers who showed increased hippocampal size attributable to intensive spatial navigational demands [19]. Using fMRI and a social-stress task, Lederbogen and colleagues (2011) found that childhood and adult urbanicity were associated with regional activation and functional coupling within the limbic system of healthy adults [20]. The same group linked urbanicity to NPSR1 [21] and to decreased gray matter volume [22]. In the case of schizophrenia, a heritable brain disorder closely tied to dopamine, studies have shown increased relative risk for those raised in an urban environment and associated with development to age 15 years [23–24]. Here, we predicted that cognitive neural function measured by our fMRI task and without explicit social stress, would be sensitive to urbanicity as suggested by prior MRI findings [22].

In addition to fMRI effects of urbanicity, we sought GxE interaction between urbanicity and dopamine genes. Dopamine genes represent critical links to cortical dopamine and its fundamental role in DLPFC function with ties to our fMRI phenotype. Additionally, stress affects dopamine [25–26], and dopamine has been linked with environmental exposures like early maternal care [27], childhood adversity [28], acute stress [29], and chronic stress [30]. While no association between dopamine and urbanicity, urbanicity and stress induces changes in cortisol, itself linked to dopamine [31]. Three dopamine genes that are expressed in prefrontal cortex and that are important mediators of prefrontal function were chosen—catechol-o-methyl-transferase gene (*COMT)*, dopamine receptor D1 (*DRD1*), and dopamine receptor D2 (*DRD2*), specifically polymorphisms *COMT* rs4680 (Val^158^Met), *DRD1* rs4532 (-48 A/G dDel), and *DRD2* rs1076560. Prior reports and a meta-analysis have linked *COMT* Val^158^Met (rs4680) to DLPFC fMRI activation [32–34]. *DRD1* and *DRD2* influence cortical DA synaptic activity and neuronal tuning [35–39]. *DRD1* rs4532 is in strong LD across the gene and DRD1 binding has been tied to fMRI memory tasks [40–41]. The *DRD1* rs4532 allele has been associated with fMRI measures of working memory tied to prefrontal activation and illnesses like schizophrenia [42–43]. *DRD2* rs1076560 minor allele carriers showed altered fMRI activation during our N-back WM task [44–46].

Given the importance of dopamine for optimal prefrontal function, we hypothesized that a main effect of childhood urbanicity would modulate the influence of *COMT*, *DRD1*, and *DRD2* polymorphisms via interaction. Based on prior associations between urban rearing and altered fMRI activation, we tested for the influence of childhood urbanicity and urbanicity-dopamine gene interactions using fMRI measures. Specifically, the N-back fMRI WM challenge known to activate a network including dorsolateral prefrontal cortex (DLPFC) [47–48] was used. We predicted that if gene-urbanicity relationships existed, they would be detected as alterations between the relationships of dopamine alleles to DLPFC activation.

## Materials and methods

### Subjects

As the *COMT* discovery sample, 124 unrelated Caucasian healthy volunteers between the ages of 18 and 55 (‘discovery’) (56 males and 68 females) were studied (Table 1). All *COMT*, *DRD1*, and *DRD2* subjects were carefully screened for medical or neurological illness, substance abuse or dependence, medications that could affect brain function, and psychiatric illness [49–50]. The availability of fMRI data, urbanicity measures and *COMT*, *DRD1*, and *DRD2* genotypes were inclusion criteria. We also tested two independent *COMT*-urbanicity replication samples of unrelated Caucasian subjects selected with the same criteria and the same methods as at discovery, a U.S. replication consisting of 137 unrelated subjects (70 males and 67 females) from our ongoing study (‘U.S. replication,’ Panel A in S1 File) and 226 unrelated subjects (99 males and 127 females) from the region of Puglia, Italy (‘Italian replication,’ Panel B in S1 File). The *DRD1/DRD2* sample was composed of 253 healthy volunteers (110 males and 143 females) combined from the discovery and US replication samples (Panel C in S1 File).

**Table 1:** Demographic characteristics and working memory performance (Discovery sample).

	**All Subjects**		**Urban**		**Town**		**Rural**		
	**(N = 124)**		**(N = 28)**		**(N = 66)**		**(N = 30)**		
**Sex**									
** Male**	56		15		31		10		
** Female**	68		13		35		20		
***COMT* Genotype**									
** Val/Val**	22		5		15		2		
** Val/Met**	60		12		30		18		
** Met/Met**	42		11		21		10		
	**Mean**	**SD**	**Mean**	**SD**	**Mean**	**SD**	**Mean**	**SD**	**P**
**Age (years)**	31.9	8.9	31.5	9.3	32.4	9.0	31.1	8.4	n.s.
**Education (years)**	17.3	2.3	17.3	1.4	16.8	2.4	18.4	2.5	0.007
**SES**	51.6	8.9	53.4	7.5	50.9	9.4	51.7	9.0	n.s.
**Childhood SES**	51.7	11.5	54.6	9.8	50.5	11.5	51.9	12.7	n.s.
**WAIS-IQ**	112.1	9.1	111.6	9.4	111.9	9.1	113.1	9.3	n.s.
**Handedness**	75.0	45.5	59.6	63.5	83.7	25.4	70.3	56.2	n.s.
**2-Back % Correct**	78.7	16.2	76.5	14.3	78.1	16.9	81.8	16.6	n.s.
**2-Back RT (msec)**	522.5	286.3	509.1	346.2	523.5	265.2	532.5	281.7	n.s.

Demographic differences across urbanicity categories at discovery. Significant differences are denoted by p values. Abbreviations: SES = socioeconomic status, WAIS-IQ = Wechsler Adult Intelligence Scale—Intelligence Quotient, handedness as measured by the Edinburgh Handedness Inventory, n.s. = non-significant.

We gathered US subjects through the National Institute of Mental Health (NIMH) with approval of the National Institutes of Health Committee for the Scientific Review of Protocols (CSRP) and the Combined Neurosciences IRB (CNS IRB). The local Institutional Review Board (Comitato Etico Locale Indipendente Azienda Ospedaliera “Ospedale Policlinico Consorziale” Bari) approved the Italian study. After complete description of the protocol and procedures, all participants gave written informed consent in accordance with the Helsinki Declaration.

### Genotyping

Subjects were genotyped for the *COMT* rs4680, *DRD1* rs4532, and *DRD2* rs1076560 single nucleotide polymorphisms (SNPs) using the 5′ exonuclease TaqMan^®^ assay and direct sequencing (Italian sample). SNP probe and primer sets were acquired as ‘Assays on Demand’ from Applied Biosystems using DNA extracted from transformed B lymphocyte cell lines using standard procedures [31]. Additional genotypes were taken from genome-wide variation data based on Illumina 550k SNP chip genotypes. Genotyping completion rate was >99% and all genotypes were in Hardy-Weinberg equilibrium (HWE p>0.5). Due to the low number of minor allele homozygotes for *DRD2* rs1076560, minor allele homozygotes and heterozygotes were collapsed. Occult differences in ancestry or genetic stratification could theoretically confound the findings since no single polymorphism acts alone independent of genetic background. Thus, stratification based on urbanicity (i.e., systematic ancestral differences between rural and urban environments) was tested in the US samples for whom we had genome-wide variation data based on Illumina 550k SNP chip genotypes. PLINK [51] was used to calculate pairwise identity-by-state (IBS) distances (‘plink –genome –ibs-test’). We also tested across these Caucasian subjects via multidimensional scaling using PLINK (MDS; ‘plink –genome –mds-plot’). In addition, probabilistic principal component analysis (pPCA) using R (‘ppca’ from pcaMethods) was performed [52] (Panel G in S1 File).

### Urbanicity

During the Structured Clinical Interview for DSM-IV [52], subjects were asked where they lived from birth through age 15. Historical census data was used to determine the population for these locations during relevant years. As elsewhere [20–22, 53], we used these population measures to assign urbanicity categories as follows: rural = 1 for <10,000 inhabitants (lowest urbanicity); town = 2 for 10,000–100,000 inhabitants (moderate urbanicity); and urban = 3 for >100,000 inhabitants (highest urbanicity). We used the maximal urbanicity in a given 5 year interval, an approach common to other MRI studies examining urbanicity [20–22,53]. We multiplied each urbanicity rating from the 5 year intervals by 5, yielding three scores. These scores were then summed to represent urbanicity across childhood (scores ranging from 15–45). We examined two other childhood urbanicity schemes that did not alter our results– one taking the maximum score from the urbanicity category where the majority of time was spent [54] and then a second that created a mean score across childhood (0–15 years old) [55]. Our method is a modification of more traditional approaches that create create composite urbanicity scores based on yearly measures between 0–15 years old [20–22,53]. We note studies that used similar 5-year intervals as ours [55–57] and even coarser measures than ours to calculate urbanicity (e.g. only urbanicity at birth to predict adult psychopathology [58–59]. Regardless, our approach remains non-traditional based on the majority of urbanicity studies. Traditional yearly measures may have a more finer-grained resolution when mapping effects of urbanicity than our 5-year intervals. Our approach to urbanicity still provides a summary measure that might correlate with traditional methods, but this remains untested.

### fMRI working memory task

Blood-oxygen-level dependent (BOLD) signal data were acquired with fMRI during a WM N-back paradigm that generates a heritable prefrontal phenotype [60–61]. Numbers from 1–4 were serially presented at set points of a diamond, one at a time every 2 seconds for 500 msec. Subjects were instructed to press buttons on a response box corresponding to the number seen “n” previously. Each subject was tested at 0- and 2-back memory loads. Therefore, each number was a probe as well as a target. A block design was used in which the 0-back (a control condition not involving WM) alternated with the 2-back task for 30 seconds each over eight repetitions. The task was presented on a screen in the MRI scanner using the software program Presentation, and it was synchronized with the start of the scanner. The screen background was a rectangle of 18 in. x 9.125 in. and the task itself was presented on rectangle of 11.75 in. x 6.5 in. Performance was measured as percent correct responses (accuracy) and subjects were excluded for excessive errors (0-back < 95% and 2-back < 25% correct or 2-back performance below chance).

### fMRI data acquisition and analysis

Whole-brain BOLD fMRI data were acquired at 3Tesla (General Electric Signa Scanners, Milwaukee, WI) using gradient-echo echo planar imaging (TR = 2000 msec, TE = 30 msec, flip angle = 90°, field of view = 24 cm, matrix = 64x64, 24 interleaved slices, 128 volumes) [62]. The fMRI images were registered to high-resolution anatomical images and analyzed using Statistical Parametric Mapping 5 (SPM5) [63] and MATLAB 7.3.0(R2006b) on a Linux operating system. For the U.S. *COMT* replication, the Italian *COMT*, and *DRD1/DRD2* samples, fMRI data acquisition and analysis were perfomed in the same way as for the *COMT* discovery sample. The data were corrected for head motion artifacts, with six motion parameters (three translational (X, Y, Z) and three rotational (pitch, roll, yaw)) used as covariates of no interest in first level analyses. The fMRI data were then anatomically normalized into a standard space Montreal Neurological Institute (MNI) template [64] using affine and nonlinear transformations, and then smoothed with an 8mm full-width at half-maximum Gaussian filter to minimize noise and to account for residual inter-subject differences. As a result, all images are reported in MNI space. Images were resampled to a 2mm isotropic voxel size and signal modelled using a boxcar convolved with the hemodynamic response function at each voxel. In addition to motion, we assessed data quality and excluded subjects based on average signal-to-noise ratio, ghosting index, signal regularity, scaled variance, slice-by-slice variance, scaled mean voxel intensity, and maximal/mean/minimal slice variance. Data with movement in any one direction (> 2mm translation or > 1.5 degrees rotation) were excluded. We did not find any significant differences in movement when comparing framewise displacement across urbanicity, genotype, or urbanicity-genotype groupings [65].

For first level analyses, the average BOLD signal from the 2-back task was contrasted with the signal from the 0-back task for each subject in order to identify the brain regions activated during the WM aspect of the task. The timings used for each of the blocks (2-back and 0-back) were from the task output, and the BOLD signal during these blocks of time was then contrasted between the 2-back and 0-back conditions. At the second level, multiple regression analyses were used to determine main effects of childhood urbanicity, dopamine genotype, as well as GxE interaction effects (dopamine gene-by-urbanicity) in one multiple regression using these three factors. These analyses were whole brain, but we report only DLPFC results as this was our primary hypothesized region for these tests. Post hoc SPM ANOVAs were performed in the discovery sample to confirm that each subgroup used in the multiple regression significantly differed from each other in the same direction found in the multiple regression—i.e., urban showed significantly greater fMRI activation than town, the latter significantly greater than rural. Sex and age were used as covariates of no interest in all analyses. For the US discovery, US replication, and the Italian replication samples, we performed the same fMRI data acquisition and analysis. Combining US discovery and US replication samples for *DRD1* and *DRD2*, we also analysed these data in the same fashion. To illustrate the COMT replication results in terms of anatomical overlap with the results from the discovery COMT-by-urbanicity interaction, regions of interest were defined from the discovery results and then applied to each replication’s activation maps using MRIcron [66–67] at a threshold of p<0.05 uncorrected for display purposes. In addition, we created a bilateral DLPFC ROI restricted to BA 9–10, 46) using the Wake Forest University (WFU) PickAtlas for further illustration of potential overlap.

For determining significant activation we used a small volume correction with family wise error correction (SVC-FWE). We created our ROIs using meta-analytically derived activation in Neurosynth [68]. Neurosynth automatically gathers coordinates from the literature using text-mining and and organizes pre- and post-analyses via key words common to many papers, such as for ‘working memory’(n = 901). The Neurosynth database is publically available (http://neurosynth.org/). Neurosynth meta-analyses are easily searchable for the papers contributing to any given meta-analysis and surfable using a 3-D graphical depiction of significant results within brain (Panel H in S1 File). Neurosynth can determine the strength of association between the search term’s coordinate measures across these studies, for example comparing studies with and without a given search term. Resultant statistics are performed on a whole brain basis. We used the reverse inference as this is recommended. Reverse inference measures can be evaluated by z-scores based on the selectivity of regional activation to working memory. Said another way, meta-analytic results link brain activity patterns with measurable cognitive states. All Neurosynth meta-anaylses are adjusted for multiple corrections at a false discovery rate of P FDR corrected <0.01.Using the key words ‘working memory,’ we identified regional peak activations using the 3-D brain representation of the meta-analysis in Neurosynth. These Neurosynth peaks were used to generate spherical ROIs for small volume correction within SPM (Panels F and H in S1 File). Neurosynth results were similar to other meta-analyses of working memory [69–71]. We analyzed the significance of activation in our study against small volume corrections using Neurosynth-based spherical ROIs in SPM8 (Panels F and H in S1 File). In summary, we analyzed significant foci of activation from our study for small volume correction using spherical ROIs based on Neurosynth (Tables 2–4, Panels F and H in S1 File). Cohen’s d were calculated in SPM using the VBM5 toolbox [72]. The VBM5 toolbox transforms SPM T maps into effect sizes, correlation coefficients, p-values, or log p-values. These effect sizes that are not explicitly defined by a primary peak within the SPM results. This statistical transformation produced images composed of clusters thst were were now surfable where voxel intensity across significant clusters was replaced by Cohen’s d. We located the maximum and range of d by surveying within each cluster. Finally, all fMRI coordinates are reported in standard MNI space.

**Table 2:** Urbanicity and *COMT* interaction effects at discovery (n = 124).

**Main effect of urbanicity**				
X	Y	Z	Region	p _SVC-FWE_
-33	45	27	L Middle Frontal Gyrus (BA 9/10)	0.03
-45	12	24	L Middle Frontal Gyrus (BA 9)	0.04
**Interaction of *COMT*-Urbanicity**				
X	Y	Z	Region	p _SVC-FWE_
-30	42	27	L Middle Frontal Gyrus (BA 9/10)	0.03
-45	12	24	L Middle Frontal Gyrus (BA 9)	0.04

Coordinates are in MNI (from MNI = Montreal Neurological Institute) space with Brodmann areas (BA) indicated in parentheses. Our threshold for statistically significant regions surviving family-wise error, small volume correction (FWE SVC) p<0.05. Abbreviations: L = left, R = right.

**Table 3:** Main effect of urbanicity and COMT-urbanicity interaction: US replication (n = 137).

**Main effect of urbanicity**				
X	Y	Z	Region	p _SVC-FWE_
-33	6	39	L Middle Frontal Gyrus (BA 9/6)	0.02
**Interaction of COMT-Urbanicity**				
X	Y	Z	Region	p _SVC-FWE_
-33	0	36	L Middle Frontal Gyrus (BA 9/6)	0.04

Coordinates are in MNI (from MNI = Montreal Neurological Institute) space with Brodmann areas (BA) indicated in parentheses. Our threshold for statistically significant regions surviving family-wise error, small volume correction (FWE SVC) p<0.05. Abbreviations: L = left, R = right.

**Table 4:** Main effect of urbanicity and *COMT*-urbanicity interaction: Italian replication (n = 226).

**Main effect of Urbanicity**				
X	Y	Z	Region	p _SVC-FWE_
42	34	36	R Middle Frontal Gyrus (BA 9)	0.02
**Interaction of *COMT*-Urbanicity**				
X	Y	Z	Region	p _SVC-FWE_
42	34	36	R Middle Frontal Gyrus (BA 9)	0.03
-48	42	16	L Middle Frontal Gyrus (BA 10/46)	0.04

Coordinates are in MNI (from MNI = Montreal Neurological Institute) space with Brodmann areas (BA) indicated in parentheses. Our threshold for statistically significant regions surviving family-wise error, small volume correction (FWE SVC) p<0.05. Abbreviations: L = left, R = right.

We also tested for differences between urbanicity groups in age, education, socioeconomic status (SES) [73], IQ, handedness (Edinburgh Handedness Inventory) [74], and N-back performance. For any demographic factor that differed between groups, analyses were repeated using it as a covariate of no interest and compared to the initial results. To further confirm that the results related to urbanicity were not confounded by socioeconomic status (SES), analyses were repeated using current and childhood SES as covariates. Additional analyses in the *COMT* discovery sample were performed to address confounding effects of current urbanicity. If individuals had moved to another urbanicity category after childhood, then the childhood measures could have represented current urbanicity (urban environment at the time of study). We investigated this possibility in two ways. First, we repeated the analyses substituting current urbanicity for childhood urbanicity and using only subjects who had changed urbanicity categories. Second, we calculated the difference between childhood and current urbanicity and added this change in urbanicity into the multiple regression design. Next, given evidence elsewhere of a potential interaction between *COMT* and sex [75–76], all fMRI analyses used sex as a covariate of no interest. In addition, the discovery data were re-analyzed in male and female subjects separately. As some cell sizes were small (although never the same cells across all three samples), we estimated effects ‘as if’ the *COMT* discovery results depended upon smaller subsamples subsumed in the multiple regression analyses at discovery and collapsed to a two-sample t test.

## Results

### Demographic and performance results

Across cohorts, neither N-back performance nor reaction time differed across *COMT*, *DRD1*, or *DRD2* genotype or urbanicity groups (Table 1). In the discovery sample, the only difference across groups in demographic or WM performance measures was for years of education. Subsequent analysis of this *COMT* discovery sample using the same multiple regression approach plus years of education as a covariate of no interest did not change the results significantly. Even though no childhood socioeconomic status (SES) differences were found, we nonetheless repeated the analysis of the discovery cohort using current and childhood SES measures as covariates of no interest and found the results unchanged. In the *COMT* US replication, no demographic differences were found across genotype groups. In the *COMT* Italian replication, a difference was found in childhood SES. Therefore, we added childhood SES as a covariate of no interest to the Italian analysis, which did not significantly change the results. For the *DRD1*/*DRD2* cohort, there was a difference across groups for years of education (p = 0.02). An analysis including education as a covariate of no interest did not significantly change the results for either *DRD1* or *DRD2*. Participants were primarily right handed, and handedness did not significantly differ between urbanicity groups in the discovery, US and Italian replications, nor *DRD1*/*DRD2* samples (Panels A-C in S1 File).

Across the three COMT samples, there were no genotype differences between urban groups using Chi-square tests at discovery (p = 0.3) or in the Italian sample (p = 0.9). A trend for genotype frequency differences in the U.S. replication sample (p = 0.06) was found, but no overall genotype frequency differences in *COMT* between urban and non-urban groups when collapsed across the three samples (p = 0.4). For *DRD1* and *DRD2* (n = 253), no differences in genotype frequencies for *DRD1* rs4532 (Chi square p = 0.89) or *DRD2* rs1076560 (Chi square p = 0.72) were found. Using the *COMT* discovery sample, no evidence was found for stratification across childhood urbanicity via IBS measures. No evidence for clustering based on urbanicity nor indication of significant heterogeneity was found either. Permutation testing for urbanicity-related IBS differences was not significant (all p > 0.3). We also failed to detect evidence for ancestry differences based on significance testing using the means and standard deviations (all p > 0.1) and scatterplots of the first nine components from probabilistic principal component analysis showed no apparent outliers enriched in either urban or rural samples (Panel G in S1 File).

### fMRI results

**Fig 1:**
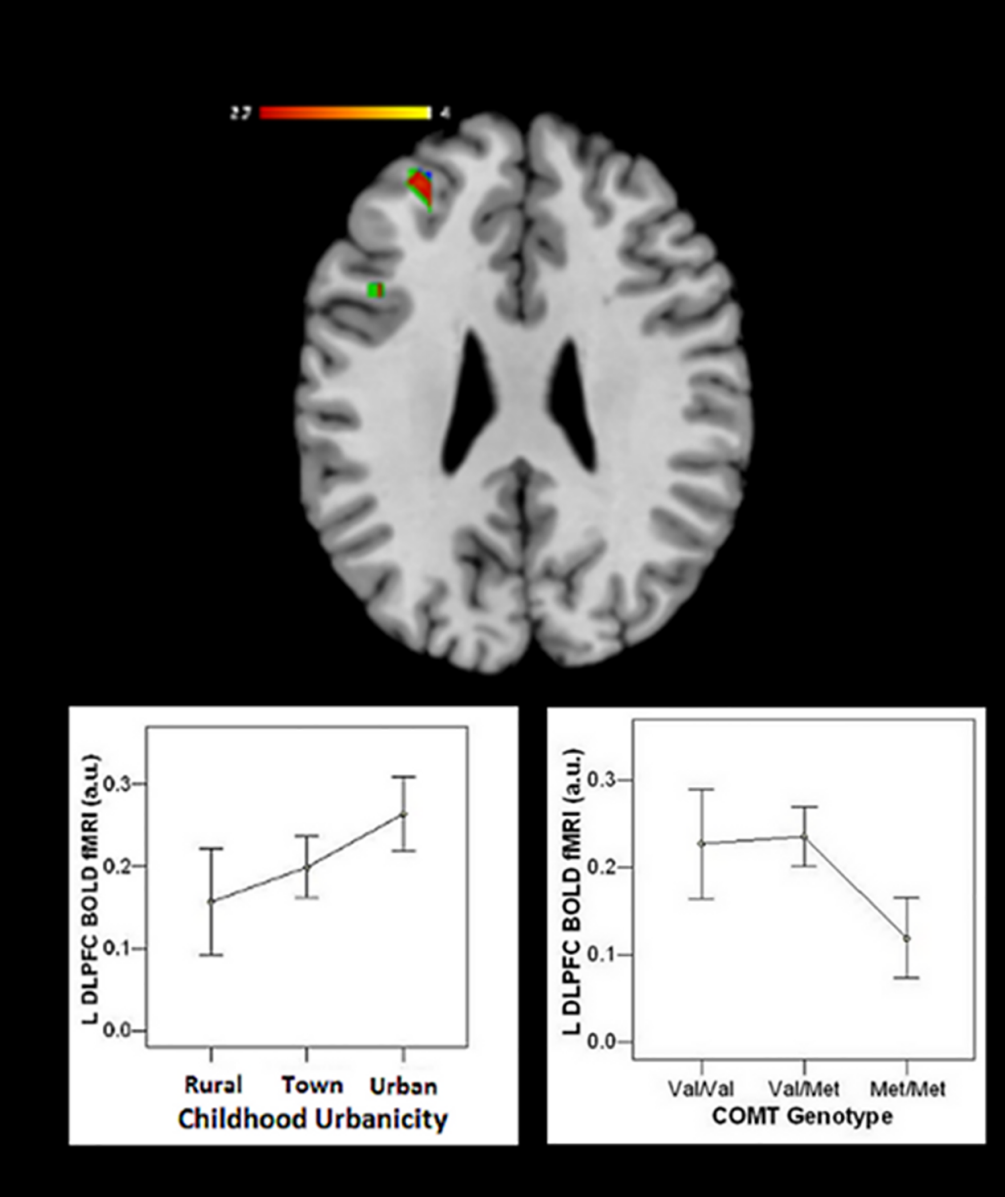
Urban upbringing and *COMT* Val/Val associated with prefrontal working memory inefficiency. In the top center image, main effects of urbanicity (green), *COMT* (blue), and their overlap (red) are presented. Graphs in the lower left (urbanicity) and lower right (*COMT* genotype) use values extracted from the imaging analysis to illustrate that both urban upbringing and *COMT* Val genotype predicted greater prefrontal activation during working memory at discovery. The fMRI activation threshold is p< 0.005 (uncorrected). Parameter estimates are graphed in arbitrary units (a.u.) (mean ± standard error). Left = Left.

**Fig 2:**
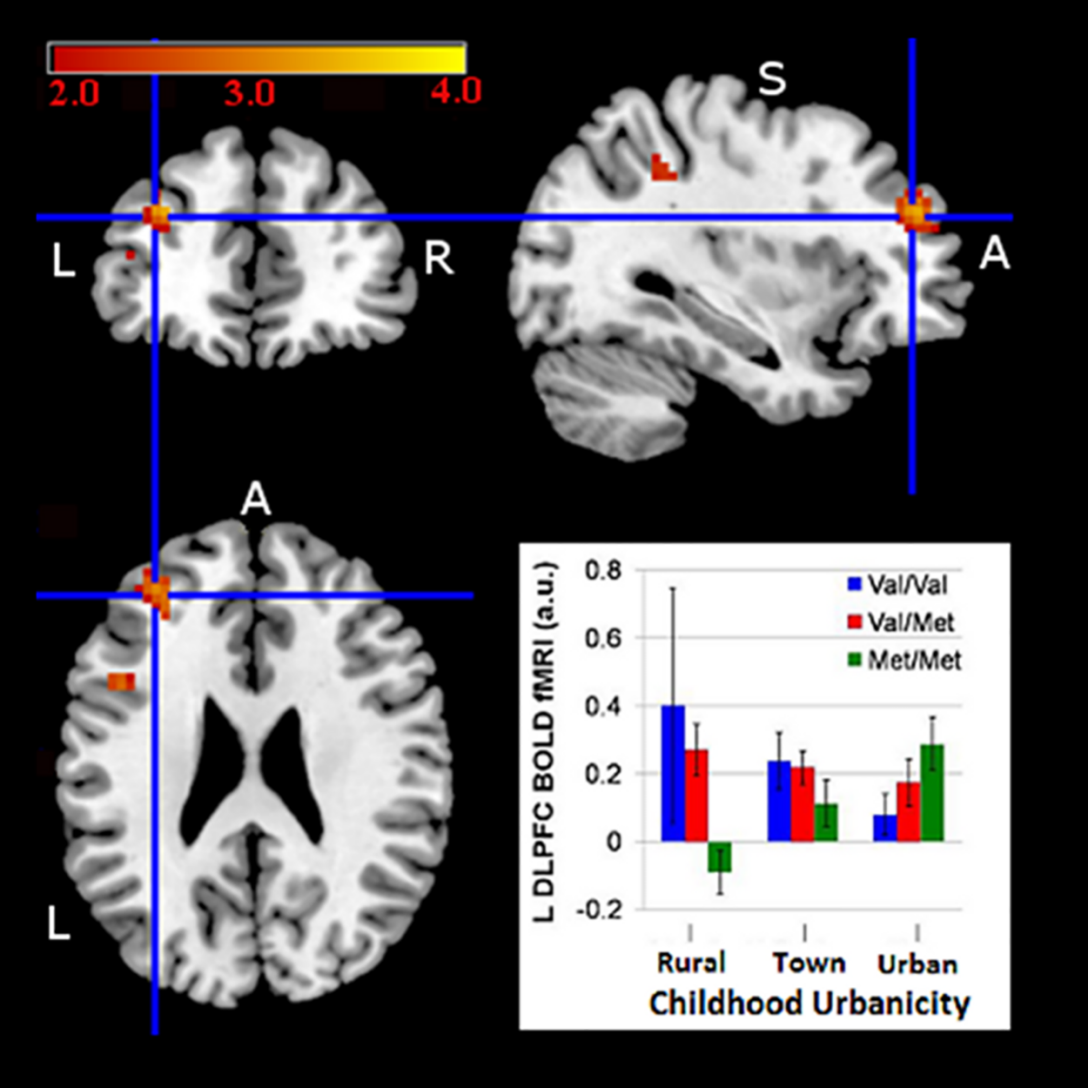
Urbanicity-by-*COMT* at discovery: Urban upbringing reversed the relationship between *COMT* and prefrontal working memory activation. Urbanicity-by-*COMT* interaction at discovery showing urban Met/Mets paradoxically over-activated relative to Val/Vals and contrasting with patterns found for rural and town. Our threshold for statistically significant regions survived family-wise error, small volume correction (FWE SVC) p<0.05. Abbreviations: L = left, R = right, X, Y, Z coordinates from MNI = Montreal Neurological Institute. BOLD graphed as mean ± 1 standard error (a.u. or arbitrary units) with the color bar above representing activation significance. Left = Left, A = anterior, L = left, R = right, S = superior.

**Fig 3:**
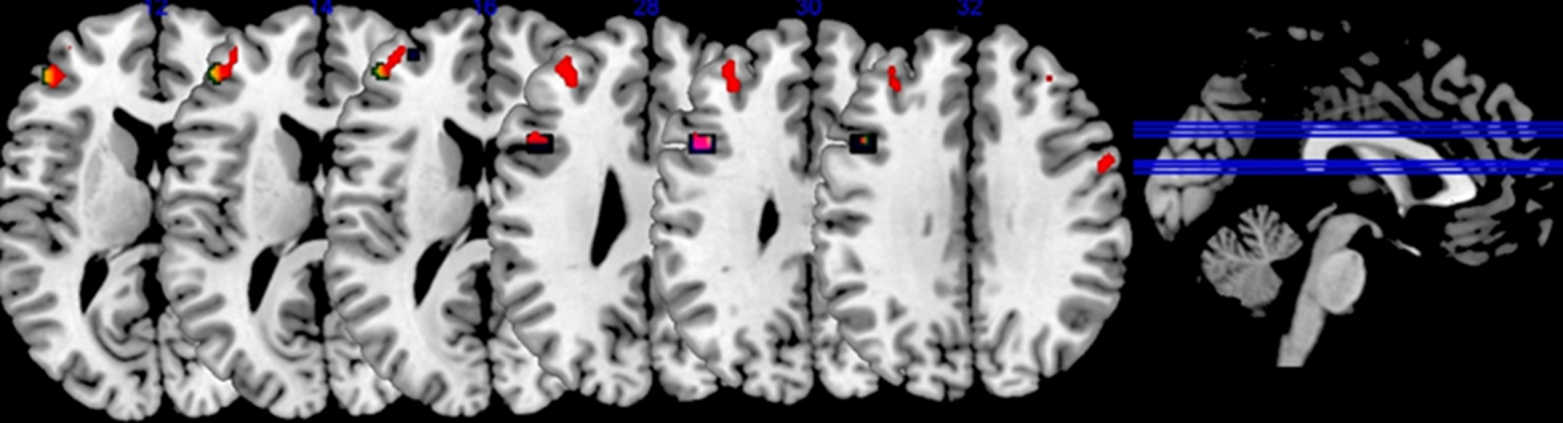
Urbanicity-by-*COMT* interaction results from discovery sample showing spatial overlap with US and Italian replications (n = 487). Areas of spatial overlap between the discovery results and those at replication. Red indicates the discovery sample, blue the US replication and yellow the Italian replication, while other colors resulted from overlap. The statistical threshold for display purposes is p <0.05 (uncorrected). Left = Left.

We found main effects of urbanicity and dopamine genes on prefrontal activation and gene-by-urbanicity interactions. In the *COMT* discovery sample, those growing up exclusively in an urban setting (n = 28) were inefficient—defined as more BOLD activation for equivalent task performance—relative to those raised in rural (n = 30) or town (n = 66) environments (Fig 1, Table 2). As elsewhere [31,62] a main effect of *COMT* genotype was found, with Val allele having the greatest DLPFC activation, i.e. being more inefficient (Fig 1). Additionally, an interaction was found between *COMT* and urbanicity in the left DLPFC (Fig 2, Table 2). Subjects raised outside of urban areas (populations <100,000) showed the well-described effect wherein Val/Val individuals were inefficient relative to Met/Met subjects. In contrast, urban-reared subjects showed a reversal of the typical *COMT* effect in left DLPFC where Met/Met individuals (n = 11 of 28) were relatively inefficient compared to Val/Val subjects (Fig 2, Table 2). In other words, urban subjects failed to show the expected prefrontal activation advantage of the Met allele. For our analyses, the opposite contrasts revealed no significant DLPFC activation of the magnitude of the above effects. Using current (i.e. adult urbanicity), neither main effects of current urbanicity nor an interaction between current urbanicity and *COMT* were found. We found activation across the three samples in a ROI comprised of bilateral BA 9,10, and 46 (Fig 3). However, within the DLPFC we did not find any activation using the opposite contrasts for effects of urbanicity, COMT, or COMT-urbanicity interactions in any analyses presented below.

Urbanicity, *COMT* genotype, and *COMT*-by-urbanicity results in left DLPFC were replicated in the two additional, independent Caucasian samples (Tables 3 and 4). We found *COMT*-by-urbanicity effects that occurred in the same locations seen at discovery, even when held to a similar statistical threshold as at discovery (Fig 3).

Moderate effect sizes for the typical *COMT* Val>Met inefficiency were found within DLPFC at discovery (d = 0.50–0.64), approaching but not meeting *COMT* meta-analytical results reported elsewhere (d = 0.73–0.91) [33]. For both childhood urbanicity and the *COMT*-urbanicity interaction, effect sizes were also moderate (d = 0.47–0.60) and (d = 0.51–0.63), respectively). We also calculated effect sizes for the two replication samples. In the US replication sample, moderate effect sizes were found for COMT (d = 0.46–063), urbanicity (d = 0.58–0.79), and the COMT-urbanicity interaction (d = 0.50–0.78). Effect sizes were smaller for the Italian replication sample—COMT (d = 0.22–0.32), urbanicity (d = 0.24–033), and the COMT-urbanicity interaction (d = 0.30–0.38).

**Fig 4:**
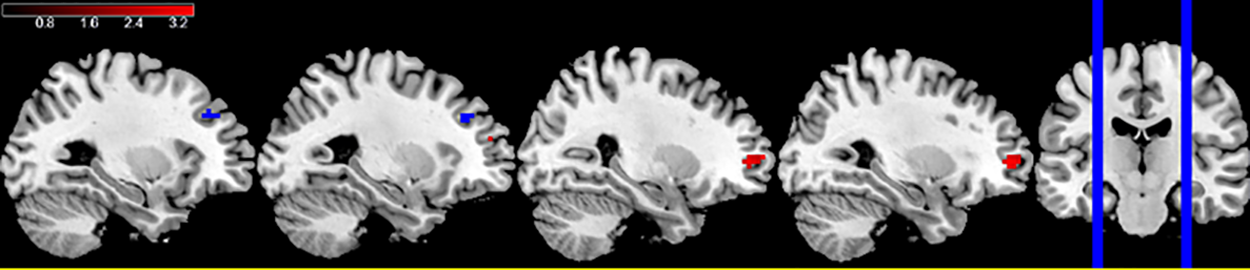
*DRD1*-by-urbanicity, *DRD2*-by-urbanicity and *COMT*-by-urbanicity interactions (n = 253). Dorsolateral prefrontal GxE interactions between dopamine genes and urbanicity. The *DRD1* (rs4532)-by-urbanicity interaction is shown in red, while the *DRD2* (rs1076560)-by-urbanicity interaction appears in blue. The statistical threshold for significance was p<0.005 (uncorrected). The color bar represents activation significance. Left = Left.

We found GxE interactions for both of *DRD1* and *DRD2* (Fig 4; Panels D-E in S1 File). A *DRD1* GxE interaction was found in bilateral DLPFC (right BA 10 [30 51 3] and left BA 9/10 [−24 48 15], both FWE SVC p<0.05; d = 0.47 and 0.39, respectively) showing an alteration of the genotype effect for urban subjects. *DRD2*-urbanicity interactions were found in left DLPFC (left BA 9/10 [−27 36 36] p = 4e-4; d = 0.40). Similar to the *COMT* interaction, moderate effect sizes for *DRD1*-by-urbanicity and *DRD2*-by-urbanicity were found (Fig 4).

### fMRI: Additional factors and post hoc analyses

In the two additional analyses to test confounding effects of current urbanicity, we found no significant changes in the results with either approach. In reanalyzing the discovery data in male and female subjects separately, we found the main effects and interactions with smaller, independent cohorts grouped by sex. In the male subjects, the *COMT*-by-childhood urbanicity effect was localized at [–33 45 24] and in the female subjects localized at [–45 15 24] and [–39 48 21].

The small cell sizes in the analyses were for urban Val/Val (n = 5) and rural Val/Val (n = 2) in the discovery sample and rural Met/Met (n = 2) in the Italian replication sample. When we estimated effects ‘as if’ the COMT discovery results depended upon smaller subsample, we found effects in the small cohort similar to those found in the overall analyses for the *COMT*-urbanicity interaction ([21 54 30]; [–36 54 21]).

## Discussion

This is a novel study in that it replicated a GxE interaction found using functional neuroimaging. The findings showed an interaction between *COMT* and childhood urbanicity on prefrontal activation, which replicated in two additional samples. Two other dopamine genes, *DRD1* and *DRD2*, were found to interact with urbanicity as well. Taken together, these results show how one’s environment during childhood can impact adult brain function, and specifically in relation to altering effects of dopamine genes.

### fMRI: Main findings and additional factors

In the case of *COMT*, the GxE involved a well-characterized and ‘human-specific’ genetic polymorphism (*COMT* Val^108/158^Met) using a heritable fMRI measure of working memory-related prefrontal information processing. Additionally, findings in the *COMT* replication samples overlapped anatomically with the discovery sample. We believe starting at any of the three *COMT* samples would have the same replication outcome. Importantly, childhood urbanicity and the GxE effects were similar for urban environments in different countries. In the initial discovery sample, no evidence was found for significant demographic or genetic confounds—neither differences in SES nor evidence of whole genome based stratification. There were no effects of adult or current urbanicity. Applicable to all dopamine gene genotype-phenotype effects, it is important to note that WM performance did not differ across groups and thus relative differences in activation should not have been affected by behavioral effects.

Interactions between childhood urbanicity and *DRD1* and *DRD2* were also found in the prefrontal cortex, suggesting that childhood urbanicity and GxE effects extend to other genes in the dopamine pathway. Analyses of the dopamine receptor genes *DRD1* and *DRD2* revealed GxE interactions with urbanicity (GxE). Although we did not have replication samples for *DRD1* and *DRD2* analyses, their effect sizes were also moderate. We conclude that an urban childhood has an effect on brain function as measured with fMRI during WM that persists into adulthood. Specifically, urban upbringing altered the relationship between fMRI activation and polymorphisms in *COMT*, *DRD1*, and *DRD2*. In terms of urban upbringing, these dopamine findings are evolutionarily interesting considering that dopamine mutations likely predate modern civilization and the emergence of large cities [77].

### Speculation about gene-by-urbanicity interactions

We speculate that the interaction with dopamine genes involves environmental changes in cortical dopamine availability. Dopamine sensitization or altered release of dopamine by environmental stress [25, 30, 78]—including GxE interactions [18]—have been proposed as mechanisms linking urbanicity and other environmental factors in a common pathway [14, 79]. Stress or other hitherto unknown variables associated with higher urbanicity could lead to suboptimal cortical dopamine levels and thus appear as changes in fMRI-to-dopamine gene relationship. As urbanicity might increase dopamine sensitization [80], some particular stress, social pressure, or even a toxic agent in urban centers may push subjects beyond optimal dopamine tone as with amphetamine administration in healthy subjects apart from urbanicity [81]. While we have hypothesized that urbanicity moderates the effects of dopamine genes, it is also possible that perhaps individuals with particular dopamine gene polymorphisms are more prone to sensitization related to urbanicity. While such details reach beyond our data, the findings warrant further study within these contexts.

Dopamine regulation for optimal working memory performance is dependent on both baseline dopamine state as well as dopamine effects with cognitive load [82]. The relationship between dopamine and performance has been described as an ‘inverted U,’ where too little or too much dopamine limits performance. *COMT*, *DRD1*, and *DRD2* are potentially involved in both baseline state and stimulated dopamine effects, suggesting the potential for multiple routes to suboptimal DLPFC function in the urban-reared. As a functional coding variant, *COMT* rs4680 effects on DLPFC activation reflect the decreased synaptic dopamine conferred by the more active *COMT* Val-associated catabolic enzyme. The shift towards suboptimal performance in the urban-reared Met-carriers fits nicely with dopamine sensitization wherein increased dopamine release in response to stress coupled with greater synaptic dopamine released by stressors, potentially like urban environments, would move urban Met-carriers beyond optimal dopamine. Such *COMT* changes relate directly to DLPFC dopamine levels, but the contribution of *DRD1* and *DRD2* polymorphisms are somewhat less clear.

D1 (dopamine receptor 1) is more abundant in DLPFC than D2 (dopamine receptor 2) and has been closely tied to working memory deficits. The neuropsychiatric disorder most affected by childhood urbanicity, schizophrenia, has been shown via PET imaging of D1 ligand binding to have altered DLPFC D1 receptor abundance. PET studies have shown something of the relationship between *COMT* rs4680 and D1 receptors [83]. Slifstein and colleagues (2008) found that the decreased synaptic dopamine in Val subjects caused an upregulation of D1 receptors in DLPFC for healthy subjects. These findings are analogous to the relationship in patients with schizophrenia between decreased dopamine and both increased D1 receptor binding and reduced working memory function [38]. *DRD1* rs4532 is in the 5’-untranslated region of this gene and has a relationship to *DRD1* expression [84]. This SNP may also be important to *DRD1* as it has been associated with dopamine-associated conditions including tardive dyskinesia in schizophrenia [85], attention deficit hyperactivity disorder (ADHD) [86], and response to stimulant medications in ADHD [87].

D2 receptors are more heavily expressed in the striatum but have associated function pre- and post-synaptically within prefrontal cortex—for example as an autoreceptor whose altered abundance could further negatively impact cortical dopamine. Any changes in D2 receptors could have altered striatal communication with or reciprocal regulation of DLPFC. However, we did not find any striatal changes associated with *DRD2* allele variation making cortically-centered changes more likely in this case. The *DRD2* intronic SNP rs1076560 alters the relative expression of D2 short and long isoforms (D2S and D2L)—increasing the D2S/D2L ratio and generating optimal D2 regulation in DLPFC [44]–that could be disrupted by *DRD2*-urbanicity interaction. *DRD1* and *DRD2* influence cortical DA synaptic activity and neuronal tuning and are tied to prefrontal cognitive functioning and memory performance, particularly D1 [32–45]. This relationship of D1 and D2, expressed as the D1/D2 ratio, represents relative counter-balancing in DLPFC regulation [38]; therefore presumably disrupted by genetic differences in urban versus non-urban upbringing. In schizophrenia for example, abnormal binding of D1 and D2 receptors have been shown, and the most efficacious medications target one or both [88–90]. In fact, *DRD2* has been associated with schizophrenia in a recent genome-wide association study [91]. Alterations in their relative abundance and function could serve to enable or magnify dopamine sensitization, as a cause for marked dopamine dysregulation via additive or multiplicative interactions, as downstream effectors of dysregulation arising from other dopamine-modulated regions (e.g. striatum), or as some combination of the above. In sum, these three genes may produce direct or indirect functional dysregulation in response to or causing altered dopamine within DLPFC for those raised in an urban environment.

## Limitations

A limitation in investigations such as ours is that urbanicity, as defined by population size, represents a nonspecific factor with effects that could be multi-determined and potentially modified by other influences such as SES and cultural perspectives. However, risk for schizophrenia based on urbanicity is not explained by other factors that could be associated with city living, including childhood infection, household crowding, obstetric complications, or SES [3, 58, 92–94]. Perhaps analogous to humans and urbanicity, a wealth of animal data based on changes in housing suggests that differences in human population density might yield changes in brain structure and function [13,95]. However, direct links between animal housing conditions and environmental aspects of city dwelling are lacking. Urbanicity may be associated with stress during pregnancy and childhood, including other sociocultural and health factors [27–30]. Interestingly, individuals with the *COMT* Val allele appear sensitive to stress-related methylation that negatively influences prefrontal efficiency [96]. As developmental stress is a major factor in neuropsychiatric disorders, childhood urban living could promote or exacerbate mental disorders via dopamine sensitivity or epigenetic mechanisms.

In comparison to the sample sizes used to explore genome-wide genotype effects, our large-for-neuroimaging samples would be small. Our thresholds without whole brain correction may have introduced false positives. However, the multiple replications across three samples and three dopamine genes using the significance thresholds as at discovery help to mitigate this concern. Effect sizes for all cohorts suggest our numbers were adequate. Our 5-year interval measure differs from the more common ‘urbanicity-by-year’ methods and could have made our analyses less sensitive to main and interactive effects. Repeating our analyses using years of residence and their urbanicity yielded the same results as our initial analyses. Gene-environment interactions are not necessarily multiplicative, but we did not detect a significant additive interaction between urbanicity and *COMT*, nor did the addition of an additive term significantly alter the analysis. While a previous study found no association between urbanicity and activation during the N-back task, those negative results in a much smaller study only applied to activation within amygdala and cingulate regions and not to activation within DLPFC [20].

## Conclusions

Based on these and other data, urbanicity is linked to quantifiable complex brain function, here working memory, that could generate novel insights for genetic studies of cognition and mental illness. Our findings concerned healthy people, but future work could extend into those neuropsychiatric populations known to have increased risk from urban living. Across three datasets and two countries for *COMT* and across three dopamine genes (*COMT*, *DRD1* and *DRD2)*, the data strongly suggest a lasting impact of an urban upbringing on aspects of DLPFC function as measured by fMRI, additionally altering the relationship with genetic background and potentially other critical aspects of brain function.

## Supporting information

S1 File Supporting Tables and Figures. **Panel A. U.S. Replication Demographic Characteristics and Working Memory Performance.** Demographic differences across urbanicity categories in the US replication sample. Significant differences are denoted by p values. Abbreviations: SES = socioeconomic status, WAIS-IQ = Wechsler Adult Intelligence Scale—Intelligence Quotient, RT = reaction time, n.s. = non-significant. **Panel B. Italian Replication Demographic Characteristics and Working Memory Performance.** Demographic differences across urbanicity categories in the Italian replication sample. Significant differences are denoted by p values. Abbreviations: SES = socioeconomic status, WAIS-IQ = Wechsler Adult Intelligence Scale—Intelligence Quotient, RT = reaction time, n.s. = non-significant. **Panel C. DRD1 and DRD2 Demographic Characteristics and Working Memory Performance.** Demographic differences across urbanicity categories for *DRD1* and *DRD2* (both n = 253). Significant differences are denoted by p values. Abbreviations: SES = socioeconomic status, WAIS-IQ = Wechsler Adult Intelligence Scale—Intelligence Quotient, RT = reaction time, n.s. = non-significant. **Panel D. Main Effect of *DRD1* and *DRD2*.** Coordinates are in MNI space with Brodmann areas (BA) indicated in parentheses. Abbreviations: L = left, R = right, MNI = Montreal Neurological Institute coordinates, SVC-FWE = small volume correction using family-wise error. **Panel E. DLPFC Activation for *DRD1*- and *DRD2*-by-Urbanicity Interaction.** Coordinates are in MNI space with Brodmann areas (BA) indicated in parentheses. Abbreviations: L = left, R = right, MNI = Montreal Neurological Institute coordinates, SVC-FWE = small volume correction using family-wise error. **Panel F. Neurosynth coordinates for creating regions of interest.** Activation coordinates and corresponding Neurosynth ROI foci used for small volume correction using family-wise error (SVC-FWE). Coordinates are in MNI (MNI = Montreal Neurological Institute) space. **Panel G. Absence of genetic stratification by urbanicity.** Exploration of differences in genetic stratification using genome-wide SNP genotypes via both multidimensional scaling (MDS) in PLINK [51] and probabilistic principal component analysis [52]. Scatterplots from the latter represent the resulting nine principal components (PC) and showing no significant differences in ancestry between urban (green) and non-urban (red) subjects (discovery + US replication). **Panel H. Use of Neurosynth to create regions of interest.** Small volume corrections were based on maxima identified using meta-analysis in Neurosynth [68] (http://neurosynth.org/analyses/terms/). Studies for Neurosynth meta-analysis were gathered and analyzed using the search terms ‘working memory.’ The cross-hairs are centered within a maxima from left dorsolateral prefrontal cortex used to create a spherical ROI to correct local statistical maxima in each of three analyses for the COMT-urbanicity interactions and two analyses for DRD1- and DRD2-urbanicity interactions. This maxima had a z-score = 6.67 with the overall image was thresholded at z-score > 5 for display purposes. Abbreviations: XYZ = coordinates in MNI standard space, D = dorsal, V = ventral, A = anterior, P = posterior, L = left, R = right.
(DOCX)
