# Supplementary material for: Interaction of childhood urbanicity and variation in dopamine genes alters adult prefrontal function as measured by functional magnetic resonance imaging (fMRI)
Source: PLoS One. 2018 Apr 10;13(4):e0195189. doi: 10.1371/journal.pone.0195189 (PMC5892884; doi:10.1371/journal.pone.0195189)

**Interaction of childhood urbanicity and variation in dopamine genes alters adult prefrontal function as measured by functional magnetic resonance imaging (fMRI)**

**S1 Supporting Information**

Jessica L. Reed^1,2,3^, Enrico D’Ambrosio^4,5^, Stefano Marenco^1^, Gianluca Ursini^4,5^, Amanda B. Zheutlin^1^, Giuseppe Blasi^5^, Barbara E. Spencer^1^, Raffaella Romano^5^, Jesse Hochheiser^1^, Ann Reifman^1^, Justin Sturm^1^, Karen F. Berman^1^, Alessandro Bertolino^5^, Daniel R. Weinberger^1,4,6^, Joseph H. Callicott^1*^

^1^ Clinical and Translational Neuroscience Branch, Division of Intramural Programs, National Institute of Mental Health, National Institutes of Health, Bethesda, Maryland, United States of America

^2^ Interdisciplinary Program in Neuroscience, Georgetown University Medical Center, Washington, District of Columbia, United States of America

^3^ Experimental Therapeutics & Pathophysiology Branch, Division of Intramural Programs, National Institute of Mental Health, National Institutes of Health, Bethesda, Maryland, United States of America

^4^ Lieber Institute for Brain Development, Johns Hopkins Medical Campus, Baltimore, Maryland, United States of America

^5^ Psychiatric Neuroscience Group, Department of Basic Medical Science, Neuroscience and Sense Organs, University of Bari Aldo Moro, Bari, Italy

^6^ Departments of Psychiatry, Neurology, Neuroscience and the McKusick-Nathans Institute of Genetic Medicine, Johns Hopkins University School of Medicine, Baltimore, Maryland, United States of America

*Corresponding author

Email: [callicottj@mail.nih.gov](mailto:callicottj@mail.nih.gov)

**Panel A:**

|  | **All Subjects** | | **Urban** | | **Town** | | **Rural** | |  |
| --- | --- | --- | --- | --- | --- | --- | --- | --- | --- |
|  | **(N=137)** | | **(N=25)** | | **(N=85)** | | **(N=27)** | |  |
| **Sex** |  |  |  |  |  |  |  |  |  |
| **Male** | 70 | | 14 | | 43 | | 13 | |  |
| **Female** | 67 | | 11 | | 42 | | 14 | |  |
| ***COMT* Genotype** |  |  |  |  |  |  |  |  |  |
| **Val/Val** | 36 | | 12 | | 18 | | 6 | |  |
| **Val/Met** | 72 | | 7 | | 50 | | 15 | |  |
| **Met/Met** | 29 | | 6 | | 17 | | 6 | |  |
|  | **Mean** | **SD** | **Mean** | **SD** | **Mean** | **SD** | **Mean** | **SD** | **p** |
| **Age (years)** | 31.5 | 10.7 | 34.0 | 10.2 | 29.9 | 10.8 | 33.9 | 10.3 | n.s. |
| **Education (years)** | 15.9 | 2.2 | 16.0 | 1.7 | 15.8 | 2.3 | 16.0 | 2.1 | n.s. |
| **Childhood SES** | 50.0 | 13.9 | 49.0 | 15.2 | 52.0 | 13.2 | 44.7 | 14.1 | n.s. |
| **WAIS-IQ** | 109.6 | 9.6 | 109.6 | 10.8 | 110.2 | 9.3 | 108.2 | 9.5 | n.s. |
| **Handedness** | 80.4 | 39.1 | 75.0 | 46.2 | 84.0 | 32.7 | 74.4 | 49.4 | n.s. |
| **2-Back % Correct** | 79.0 | 18.1 | 75.0 | 18.1 | 79.9 | 18.0 | 79.6 | 18.3 | n.s. |
| **2-Back RT (msec)** | 541.4 | 341.0 | 587.5 | 268.9 | 565.4 | 377.5 | 422.6 | 247.5 | n.s. |

**Panel B:**

|  | **All Subjects** | | **Urban** | | **Town** | | **Rural** | |  |
| --- | --- | --- | --- | --- | --- | --- | --- | --- | --- |
|  | **(N=226)** | | **(N=71)** | | **(N=138)** | | **(N=17)** | |  |
| **Sex** |  |  |  |  |  |  |  |  |  |
| **Male** | 99 | | 36 | | 58 | | 5 | |  |
| **Female** | 127 | | 35 | | 80 | | 12 | |  |
| ***COMT* Genotype** |  |  |  |  |  |  |  |  |  |
| **Val/Val** | 73 | | 22 | | 45 | | 6 | |  |
| **Val/Met** | 116 | | 39 | | 68 | | 9 | |  |
| **Met/Met** | 37 | | 10 | | 25 | | 2 | |  |
|  | **Mean** | **SD** | **Mean** | **SD** | **Mean** | **SD** | **Mean** | **SD** | **p** |
| **Age (years)** | 26.1 | 6.4 | 26.3 | 5.6 | 26.1 | 6.8 | 24.9 | 5.7 | n.s. |
| **Childhood SES** | 39.2 | 17.1 | 44.9 | 15.8 | 37.1 | 17.4 | 31.8 | 14.2 | 0.001 |
| **WAIS-IQ** | 109.0 | 12.6 | 111.1 | 10.2 | 107.8 | 13.5 | 109.2 | 13.6 | n.s. |
| **Handedness** | 74 | 42 | 72 | 40 | 77 | 37 | 51 | 70 | n.s. |
| **2-Back % Correct** | 78.2 | 19.1 | 81.3 | 17.8 | 76.9 | 19.7 | 74.6 | 19.4 | n.s |
| **2-Back RT (msec)** | 537.9 | 265.7 | 499.9 | 272.9 | 558.9 | 262.9 | 526.5 | 254.0 | n.s |

**Panel C**:

|  | **All Subjects** | | **Urban** | | **Town** | | **Rural** | |  |
| --- | --- | --- | --- | --- | --- | --- | --- | --- | --- |
|  | **(N=253)** | | **(N=48)** | | **(N=151)** | | **(N=54)** | |  |
| **Sex** |  |  |  |  |  |  |  |  |  |
| **Male** | 110 | | 26 | | 66 | | 18 | |  |
| **Female** | 143 | | 22 | | 85 | | 36 | |  |
| ***DRD1* Genotype** |  | |  | |  | |  | |  |
| **T/T** | 92 | | 16 | | 57 | | 19 | |  |
| **C/T** | 135 | | 25 | | 80 | | 30 | |  |
| **C/C** | 26 | | 7 | | 14 | | 5 | |  |
| ***DRD2* Genotype** |  | |  | |  | |  | |  |
| **C/C** | 185 | | 33 | | 115 | | 37 | |  |
| **A/C+A/A** | 68 | | 15 | | 36 | | 17 | |  |
|  | **Mean** | **SD** | **Mean** | **SD** | **Mean** | **SD** | **Mean** | **SD** | **p** |
| **Age (years)** | 31.5 | 9.6 | 30.8 | 8.8 | 31.5 | 10.0 | 31.8 | 9.4 | n.s. |
| **Education (years)** | 16.7 | 2.3 | 16.8 | 2.0 | 16.3 | 2.3 | 17.3 | 2.4 | 0.02 |
| **Childhood SES** | 47.7 | 18.1 | 49.5 | 18.0 | 47.3 | 18.2 | 44.1 | 18.1 | n.s. |
| **WAIS-IQ** | 106.0 | 22.3 | 104.6 | 24.3 | 106.5 | 21.6 | 106.2 | 22.7 | n.s. |
| **Handedness** | 71.4 | 49.6 | 66.4 | 58.8 | 74.5 | 42.1 | 67.17 | 59.8 | n.s. |
| **2-Back % Correct** | 77.4 | 18.4 | 75.6 | 20.6 | 78.3 | 17.7 | 76.1 | 19.3 | n.s. |
| **2-Back RT (msec)** | 495.6 | 256.6 | 499.5 | 263.4 | 488.6 | 256.2 | 511.9 | 255.5 | n.s. |

**Panel D:**

| **DRD1** | | | | | | |
| --- | --- | --- | --- | --- | --- | --- |
| X | Y | Z | Region | p | T | p _SVC-FWE_ |
| 42 | 42 | 3 | R Middle Frontal Gyrus (BA 9) | 4e-4 | 3.39 | 0.02 |
| **DRD2** | | | | | | |
| X | Y | Z | Region | p | T | p _SVC-FWE_ |
| -30 | 36 | 36 | L Middle Frontal Gyrus (BA 9) | 1e-4 | 3.70 | 0.04 |

**Panel E:**

| **DRD1 x Urbanicity** | | | | |
| --- | --- | --- | --- | --- |
| X | Y | Z | Region | p _SVC-FWE_ |
| 48 | 45 | 3 | R Middle Frontal Gyrus (BA 10) | 0.006 |
| 48 | 9 | 36 | R Middle Frontal Gyrus (BA 9) | 0.04 |
| **DRD2 x Urbanicity** | | | | |
| X | Y | Z | Region | p _SVC-FWE_ |
| -30 | 36 | 36 | L Middle Frontal Gyrus (BA 9) | 0.01 |

**Panel F:**

| **Sample** | **Activation Coordinates** | **Neurosynth ROI Foci** |
| --- | --- | --- |
| Discovery | -33 45 27 | -40 42 26 |
|  | -45 12 24 | -46 24 26 |
|  | -30 42 27 | -40 42 26 |
| US Replication | -33 0 36 | -44 2 36 |
|  | -33 6 39 | -36 6 40 |
| Italian Replication | 42 34 36 | 42 34 32 |
|  | -48 42 16 | -44 36 20 |

**Panel G:**


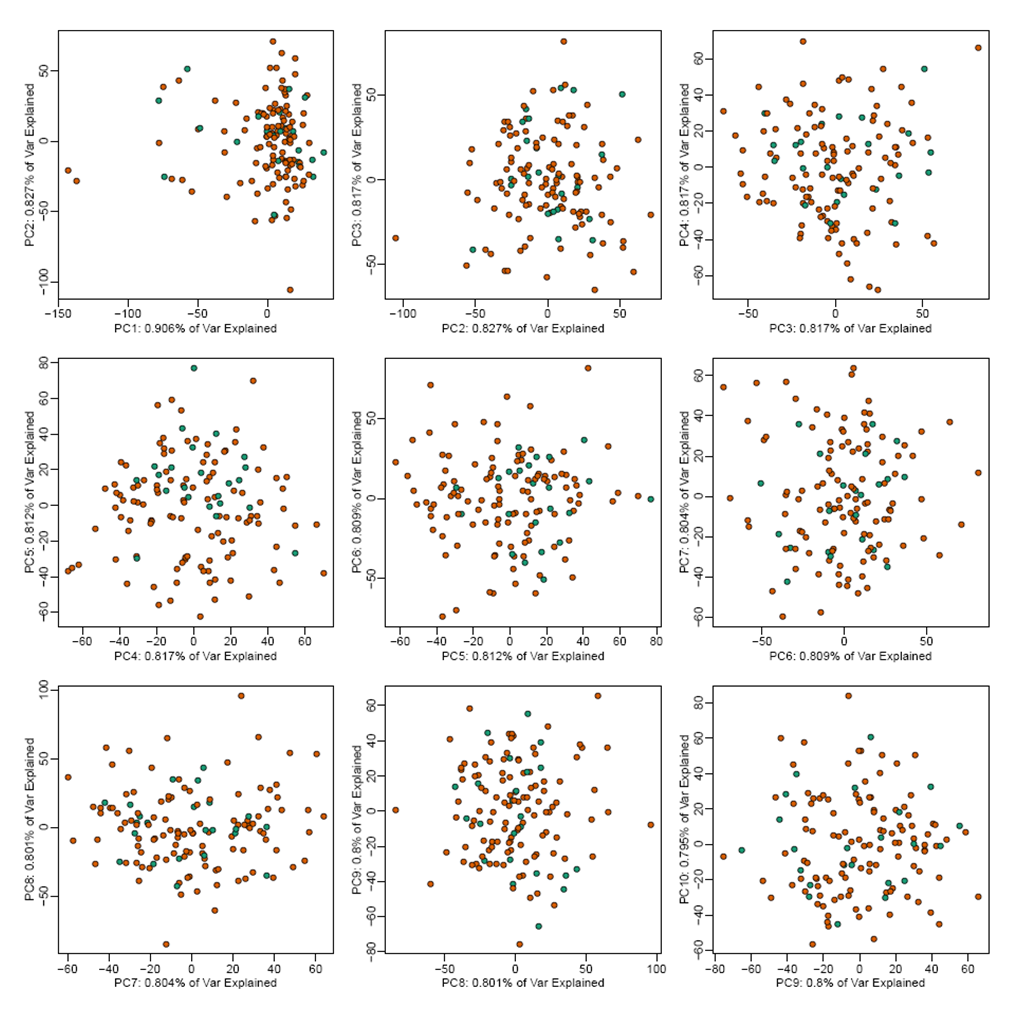


**Panel H:**


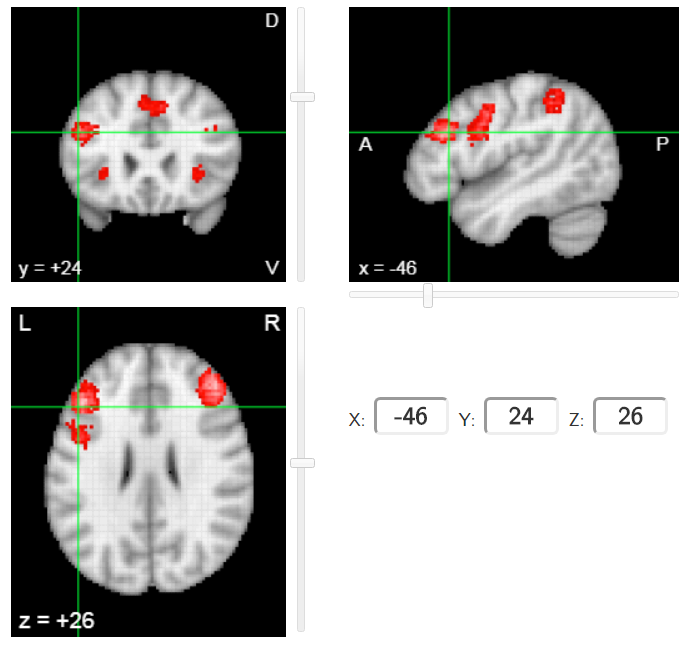

Supplement: S1 File — Panel A. U.S. Replication Demographic Characteristics and Working Memory Performance. Demographic differences across urbanicity categories in the US replication sample. Significant differences are denoted by p values. Abbreviations: SES = socioeconomic status, WAIS-IQ = Wechsler Adult Intelligence Scale—Intelligence Quotient, RT = reaction time, n.s. = non-significant. Panel B. Italian Replication Demographic Characteristics and Working Memory Performance. Demographic differences across urbanicity categories in the Italian replication sample. Significant differences are denoted by p values. Abbreviations: SES = socioeconomic status, WAIS-IQ = Wechsler Adult Intelligence Scale—Intelligence Quotient, RT = reaction time, n.s. = non-significant. Panel C. DRD1 and DRD2 Demographic Characteristics and Working Memory Performance. Demographic differences across urbanicity categories for DRD1 and DRD2 (both n = 253). Significant differences are denoted by p values. Abbreviations: SES = socioeconomic status, WAIS-IQ = Wechsler Adult Intelligence Scale—Intelligence Quotient, RT = reaction time, n.s. = non-significant. Panel D. Main Effect of DRD1 and DRD2. Coordinates are in MNI space with Brodmann areas (BA) indicated in parentheses. Abbreviations: L = left, R = right, MNI = Montreal Neurological Institute coordinates, SVC-FWE = small volume correction using family-wise error. Panel E. DLPFC Activation for DRD1- and DRD2-by-Urbanicity Interaction. Coordinates are in MNI space with Brodmann areas (BA) indicated in parentheses. Abbreviations: L = left, R = right, MNI = Montreal Neurological Institute coordinates, SVC-FWE = small volume correction using family-wise error. Panel F. Neurosynth coordinates for creating regions of interest. Activation coordinates and corresponding Neurosynth ROI foci used for small volume correction using family-wise error (SVC-FWE). Coordinates are in MNI (MNI = Montreal Neurological Institute) space. Panel G. Absence of genetic stratification by u [file pone.0195189.s001.docx]
